# Clinical efficacy and safety of warm acupuncture in the treatment of type 2 diabetic kidney disease: A protocol of a randomized controlled trial

**DOI:** 10.1097/MD.0000000000032034

**Published:** 2022-12-02

**Authors:** Yuancheng Gao, Yue Ji, Yulin Song, Rui Gong, Cheng Chen, Hongbo Chen

**Affiliations:** a Zhejiang Chinese Medical University, Hangzhou, China; b Tianjin University of Traditional Chinese Medicine, Tianjin, China; c The First Affiliated Hospital of Zhejiang Chinese Medical University (Zhejiang Provincial Hospital of Chinese Medicine), Hangzhou, China.

**Keywords:** protocol, randomized controlled trial, type 2 diabetic kidney disease, warm acupuncture

## Abstract

Type 2 diabetic kidney disease (DKD) is one of the most common and harmful chronic complications in clinical practice, and there is no reliable and targeted treatment plan at present. As a classic complementary and alternative therapy, evidence have shown that warm acupuncture has advantages in the treatment of type 2 DKD. However, there is still a lack of high-quality and long-term follow-up randomized controlled trials of warm acupuncture in the treatment of type 2 DKD.

**Methods:**

This is a prospective randomized controlled trial to investigate the efficacy and safety of warm acupuncture in the treatment of type 2 DKD. Participants will be randomly assigned in a 1:1 ratio to either the treatment group (treated with conventional Western medicine) or the control group (treated with warm acupuncture added on the basis of the control group). Both groups will receive 12 weeks of treatment followed by 24 weeks of follow-up. Observation indicators include: 24-hour urinary protein quantification, kidney function, TCM syndrome score and adverse reactions. Finally, SPSS21.0 software will be used to analyze the data.

**Discussion:**

This study will evaluate the efficacy and safety of warm acupuncture in the treatment of DKD, and the results of this trial will provide clinical evidence for the treatment of type 2 DKD.

**Trial registration:**

The TCTR identification number is TCTR20221104004.

## 1. Introduction

Diabetic kidney disease (DKD) is characterized by renal microangiopathy, some patients develop to end-stage renal disease and even need to rely on renal replacement therapy to maintain life.^[1]^ The existing clinical data show that the number of DKD patients worldwide is about 400 million and is expected to be about 592 million by 2035, accounting for about 8% to 10% of the global population. DKD has become one of the important pathogenic factors leading to end-stage renal disease, which seriously threatens the life safety of patients.^[2]^ Therefore, early control of the further development of the disease is extremely important.^[3]^ DKD has become a global public health problem, and there is no targeted treatment at present.^[4]^ There have been many studies on DKD in Western medicine, and the treatment methods have been constantly improving, mainly including conservative treatment such as controlling blood glucose, blood pressure and blood lipids, and renal replacement therapy for end-stage renal disease.^[5]^ However, it is still not ideal in reducing complications, preventing the deterioration process and long-term survival rate.^[6]^ Therefore, how to prevent its further development and improve clinical efficacy has always been the focus of research in the field of DKD, and finding a safe and effective complementary or alternative therapy is also the current trend.^[7]^

Acupuncture and moxibustion are classic complementary and alternative treatments in China.^[8]^ Previous studies have shown that both acupuncture and moxibustion have positive effects on DKD. Acupuncture can reduce the level of proteinuria in type 2 DKD,^[9]^ and moxibustion can reduce the level of serum NO and delay the progress of type 2 DKD.^[10]^ Warm acupuncture is the combination of acupuncture and moxibustion, which combines the advantages of acupuncture and moxibustion and exert a warm effect and the function of acupuncture at the same time.^[11]^ The latest clinical studies have shown that warm acupuncture has a positive effect on improving renal function in patients with DKD.^[12]^ However, there are some problems in this study, such as non-rigorous randomized method, small sample size, and lack of follow-up, which also lead to low evidence strength of research results and limit the application of warm acupuncture in type 2 DKD. Therefore, we will evaluate the clinical efficacy of warm acupuncture in the treatment of type 2 DKD through this randomized controlled trial.

## 2. Materials and methods

### 2.1. Study design

**Figure 1.**
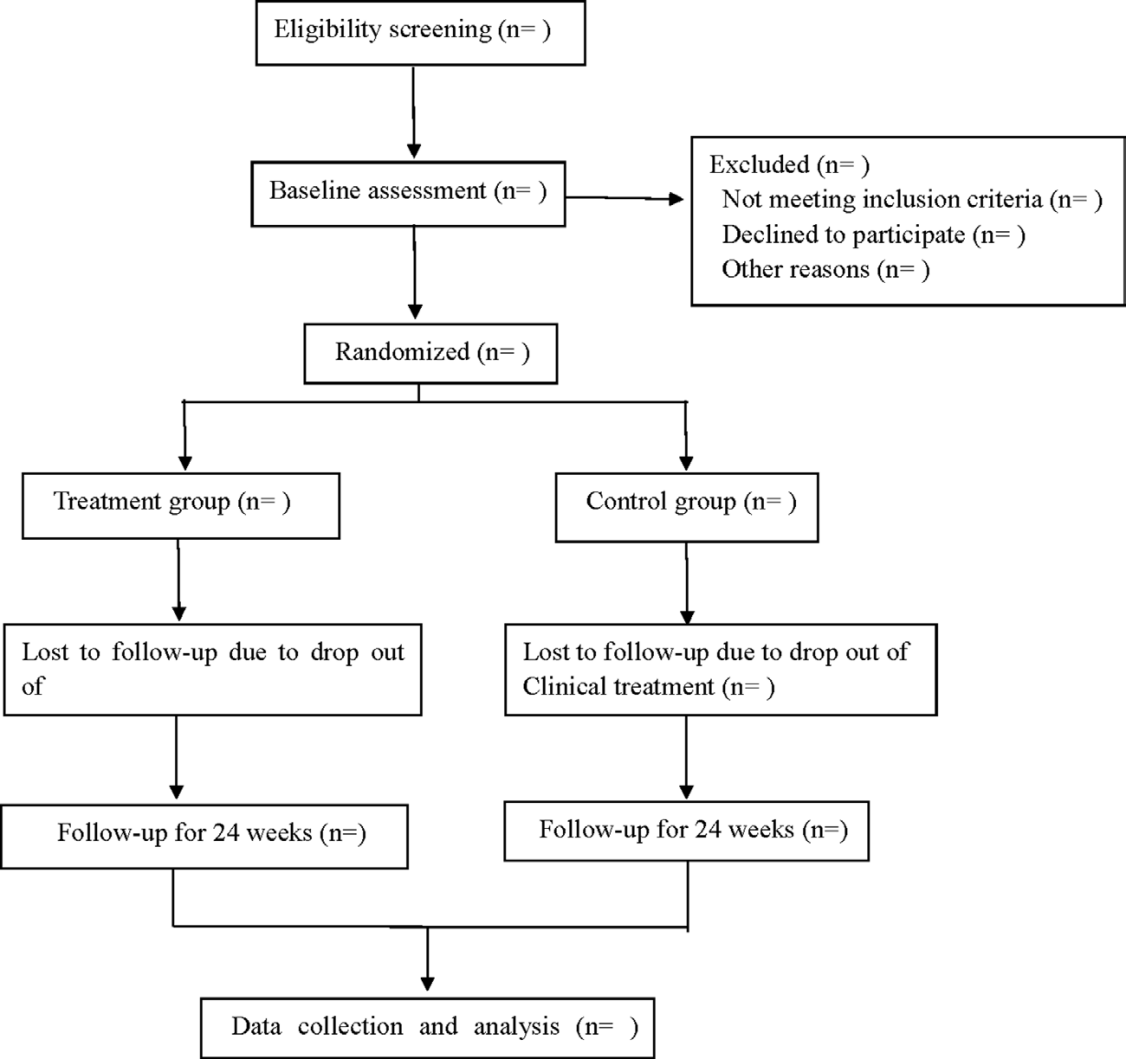
Flow diagram.

This is a prospective randomized controlled trial to investigate the efficacy and safety of warm acupuncture in the treatment of type 2 DKD. The patients who meet the criteria will be randomly divided into the treatment group and the control group. According to the recommendations of clinical guidelines, the 2 groups of patients will be treated with conventional Western medicine, and the treatment group will be treated with warm acupuncture in addition, follow up for 24 weeks after 12 weeks of continuous treatment. Flow diagram is shown in Fig. 1, and study schedule is shown in Table 1. This experiment will follow the Standards for Reporting Interventions in Clinical Trials of Acupuncture^[13]^ and the comprehensive trial report standard.^[14]^ The study will be completed under the guidance of Standard Protocol Items: Recommendations for Interventional Trials (SPIRIT checklist) 2013 statement.

**Table 1:** Study schedule.

Stage project	Screening period	Treatment period		Follow-up	
	Baseline	8 wk	12 wk	12 wk	24 wk
Record fill	√				
Fulfill inclusion criteria and exclusion criteria	√				
Sign informed consent	√				
Random allocation	√				
Treatment	√	√	√		
Effectiveness observation					
24-hr urinary protein quantification	√	√	√	√	√
Kidney function	√	√	√		√
The TCM Syndrome score	√	√	√		√
Safety assessments					
Routine blood tests	√	√	√		√
Blood test and urinalysis	√	√	√		√
Liver function	√	√	√		√
Electrocardiographic	√	√	√		
Record of adverse event		√	√	√	√

### 2.2. Ethics and registration

The study protocol complied with the Declaration of Helsinki and Ethical Guidelines for Clinical Research. This study has been reviewed by the Clinical Research Ethics Committee of our hospital and registered on the Thai Clinical Trials Registry (TCTR) (registration number: TCTR20221104004). All patients will be informed of the study plan and potential risks and will not be enrolled until they have provided written informed consent. They will be free to discontinue or withdraw from the trial at any time during the course of the study.

### 2.3. Patients

#### 2.3..1. Diagnostic criteria.

Type 2 diabetes will be defined by the American Diabetes Association criteria.^[15]^ The diagnosis of DKD will be based on the *National Kidney Foundation Kidney Disease Outcomes Quality Initiative Guidelines*.^[16]^

#### 2.3..2. Inclusion criteria.

(1) Meet the diagnostic criteria of type 2 DKD;
(2) Age ≥ 18 years old and ≤ 70 years old, regardless of gender;
(3) With the 24-hour urinary protein quantification was 0.5 to 3.5 g;
(4) With serum creatinine < 265μmol/L (3 mg/dL);
(5) Patients voluntarily participated in the trial and signed informed consent.

#### 2.3..3. Exclusion criteria.

(1) Nephropathy due to hypertension or other diseases;
(2) Complicated with severe complications and other serious organ dysfunction;
(3) Complicated with severe heart disease (NYHA class 3 or above, severe arrhythmia), abnormal liver function (alanine aminotransferase, aspartate aminotransferase ≥ 1.5 times the upper limit of normal value), hematopoietic system diseases (moderate to severe anemia);
(4) Complicated with severe infection, diabetic ketoacidosis, hyperkalemia;
(5) Complicated with severe mental illness;
(6) Pregnant or lactating women;
(7) Patients with a history of allergy to one of the drugs used in the study;
Subjects participating in clinical trials of other drugs.

#### 2.3..4. Eliminating standard.

(1) Patients with severe adverse events and/or severe complications which will be not suitable for further trial;
(2) Patients’ poor compliance affected the judgment of the results;
(3) The patient’s condition progressed rapidly during the treatment and the treatment regimen needed to be changed;
(4) The subject requested to withdraw from the trial for any reason.

### 2.4. Sample size

The sample size of this study was calculated based on the change in 24-hour urinary protein quantification (the difference in the standard deviation of the mean value of 24-hour urinary protein quantification before and after treatment) after 12 weeks of treatment, which was 0.87 ± 0.41 in the treatment group according to the results of the pilot study, and the control group was 0.65 ± 0.33. PASS15.0 software was used to estimate the sample size and set α = 0.05, β = 0.10. Finally, 37 participants are needed in each group, and the dropout rate was estimated to be 10%. The total required sample size will be 84 cases, 42 cases in each group.

### 2.5. Randomization

Eligible patients will be randomly assigned, in a 1:1 ratio, to the treatment group or the control group by means of a central web-based randomization tool. Random numbers will be generated by an independent data statistician using SAS 9.3 software (SAS Institute, Cary, NC). The researcher will enter the patient information on the computer and will be assigned a random number based on which the grouping will be completed. Efficacy assessors will be unaware of the study-group protocol, and data statisticians have no role in the design or conduct of the study.

### 2.6. Interventions

Basic treatment: treatment protocols will be developed based on the basic treatment principles established by the *2012 American Diabetes Association Practice Guidelines*,^[7,17]^ as follows: provision of health education, such as diet and exercise guidance; glycemic control; biguanides will be used in patients with obesity and/or eGFR higher than 60 mL/min/1.73 m^2^; nonobese patients and/or patients with eGFR below 60 mL/min/1.73 m^2^ will receive glinide drugs. When blood glucose control is not ideal, choose 2 oral drugs or insulin supplementation. Hemoglobin A1c (HbA1c) will be controlled below 9.0%; blood pressure control: angiotensin-converting enzyme inhibitors/angiotensin receptor blockers will be recommended. For patients whose blood pressure cannot be controlled to 140/90 mm Hg with angiotensin-converting enzyme inhibitors/angiotensin-receptor blockers, nondihydropyridine calcium channel blockers will be added, and diuretics and/or beta blockers will be added. Blood pressure will be controlled to 140/90 mm Hg or less in all patients; blood lipid control: select statins or fibrates based on blood lipid of patients.

Warm acupuncture treatment: patients will be in a sitting position, and 75% alcohol will be used to disinfected the acupuncture site. The acupuncture points are Shenshu (BL 23), Sanyinjiao (SP 6), Taixi (KI 3), Xuehai (SP 10), Pishu (BL 20), Zusanli (ST 36) and Guanyuan (CV 4), acupoint positioning is based on the positioning standards in the *National Standards of the People’s Republic of China*.^[18]^ Disposable stainless steel acupuncture needles (0.25mm × 40mm, Suzhou Hualun Medical Appliance Co., Ltd, Suzhou, China) will be used and operated by an acupuncture-moxibustion practitioner with 3 years of clinical experience. After arrival of qi, a 27-mm piece of moxa stick (Nanyang Baicaotang Co., Ltd, China, size: 18 mm × 27 mm) will be added to the handle of the needle. The bottom of the moxa stick is about 20 mm away from the skin, and the pad is separated by a small piece of paper. The bottom of the moxa section will be ignited, and the treatment will be finished after the moxa sticks was burned out. The treatment will be given for 30 minutes each time, once every other day.

The patients in the treatment group will receive basic treatment combined with warm acupuncture, while the patients in the control group only receive basic treatment. Both groups will continue to receive standard treatment for 12 weeks.

### 2.7. Outcome indicators

#### 2.7..1. Primary outcome indicator.

24-hour urinary protein quantification, which will be measured at baseline, weeks 8 and 12 of treatment, and weeks 12 and 24 of follow-up.

#### 2.7..2. Secondary outcome indicators.

1. Kidney function, which will be measured at baseline, weeks 8 and 12 of treatment, and weeks 24 of follow-up.
2. The TCM Syndrome score, which is a comprehensive record of symptoms associated with DKD, includes blurred vision, turbid urine, fatigued spirit, lack of strength, tinnitus, dizziness, spontaneous sweating, night sweats, constipation, soreness of the low back and knees, soreness of the low back and knees. At baseline, weeks 8 and 12 of treatment, and weeks 24 of follow-up, the researchers will evaluate each of the 4 levels according to the above content, as detailed in Table 2.
3. Safety assessments, including routine blood tests, urinalysis tests, liver function, and electrocardiographic results. These measures will be collected at baseline, 8 and 12 weeks of treatment, and 24 weeks of follow-up. Patients will be asked to record any unwell symptoms that occur during the study, and details of all adverse events will be recorded in a case report form.

**Table 2:** TCM symptom scoring standard.

TCM symptoms	Scoring criteria
Blurred vision	0 None
	2 The vision is not clear
	4 Blurred vision relatively difficult to distinguish objects
	6 Blurred vision difficult to distinguish objects
Turbid urine	0 None
	2 Proteinuria ±~+
	4 Proteinuria ++
	4 Proteinuria +++
Lack of strength	0 None
	1 Loss of energy after tiredness, easy recovery after rest, can insist on light manual labor
	2 Mentally tired, difficult to recover after rest, barely insist on daily work
	3 Mentally tired, unable to recover after rest, unable to persist in daily work
Tinnitus	0 None
	1 Occasional tinnitus, low ringing, intermittent
	2 Intermittent tinnitus, such as cicadas, low in the day and loud at night, can be reduced by pressing
	3 Persistent tinnitus, such as tide, continuous attacks, not reduced
Dizziness	0 None
	1 Occasional dizziness
	2 Dizziness often occurs and is tolerable
	3 Persistent dizziness
Spontaneous sweating	0 None
	1 Skin tide
	2 Wet skin
	3 Sweat out
Constipation	0 None
	1 Head sweating, occasional
	2 The chest and back are wet and recurring
	3 The whole body is wet like washing, often appears
Soreness of the low back and knees	0 None
	1 Occasionally, worse after working, can sit for a long time
	2 Often feel weak and weak in the waist, weak legs, and not sitting for a long time
	3 Back and knee soreness, unable to sit and walk
Thirst with liking for fluids	0 None
	1 Thirst, relieved after drinking water, increase daily drinking water by more than 1/2 times
	2 Thirst is obvious, drink less than 4000ml daily, or increase by 1–2 times
	3 Dry mouths, no relief after drinking water, daily drinking water volume > 4000 mL, or increase > 1 time

### 2.8. Data management and quality control

Data will be collected throughout the study and recorded in a case report form Any modification of the study protocol will be requested in advance and reviewed and approved by the ethics committee of our hospital. All data will be stored in an independent storage room to ensure data confidentiality and reliability. A Data and Safety Monitoring Board will be established. The members of the Data and Safety Monitoring Board include physicians, trial-methods experts, clinical pharmacists, statisticians, and members of the ethics committee. They will perform risk assessment and safety analysis procedures according to termination conditions. Access to the database will be restricted to researchers on this research team. The information of the subjects will not be disclosed and shared without the written permission of the subjects.

### 2.9. Statistical analysis

We will use excel to establish a database, and the outcome data will be statistically analyzed by the full analysis set and per-protocol set. Safety evaluation data will be based on the safety set. The statistical evaluation of full analysis set will follow the intention-to-treat principle. In this study, SPSS21.0 (IBM Company, New York, NY) software will be used for data analysis. The count data will be described by frequency percentage (n (%)) and a Chi-squared test will be used. Measurement data will be described by mean ± standard deviation ($\bar{x}$ ±s) or interquartile range M (P25, P75). One-way analysis of variance will be used for normal distribution comparison between groups, paired sample *t* test will be used for intra-group comparison, and Wilcoxon rank sum test will be used for skewed distribution. *P* < .05 is considered statistically significant.

## 3. Discussion

DKD is a serious microvascular complication, which is caused by long-term diabetes or poor blood glucose control, and is also an important factor in the death of diabetic patients.^[19]^ The pathogenesis of DKD is complex, and the early symptoms are occult. Therefore, clinical attention should be paid to the treatment and control of DKD.^[20]^

The pathogenesis of DKD is complicated. In recent years, the theory of autophagy has been widely used in the study of metabolic diseases, and it has been found that it is closely related to the damage of target organs. Autophagy is an important life phenomenon of living organisms; however, if autophagy is abnormally activated due to environmental stress and other factors, its autophagy expression beyond a certain physiological range will in turn cause damage to target cells, thus causing damage to target organs. The occurrence of diabetic kidney damage is closely related to the abnormal activity of autophagy and directly related to the degree of urinary protein.^[15]^ Western medicine treatment is mainly aimed to control the risk factors and further control the progress of the disease, but the clinical efficacy is still not satisfied.^[21]^

Studies have confirmed that the bidirectional regulation of acupuncture and moxibustion has something in common with the bidirectional regulation of autophagy expression. Acupuncture and moxibustion can effectively reduce urinary microprotein excretion, alleviate kidney injury and improve renal function in patients with early DKD.^[21,22]^ Warm acupuncture is a further development of acupuncture, clinical studies have shown which can reduce blood viscosity, improve microcirculation, regulate immune function, and inhibit platelet aggregation.^[23]^ Although evidence has shown that warm acupuncture is beneficial for patients with DKD, whether it can be used as a complementary or alternative treatment regimen for DKD remains controversial. We will use this study to investigate the clinical efficacy of warm acupuncture in the treatment of DKD, which is beneficial for both patients and clinical decision makers.

There are also some shortcomings in this study: due to the factors of treatment methods, this study could not achieve double blindness, which may have some impact on the study results; at the same time, there may be population regionalization in the study.

## Author contributions

**Conceptualization:** Yuancheng Gao and Yue Ji.

**Data curation:** Yulin Song and Rui Gong.

**Formal analysis:** Cheng Chen and Hongbo Chen.

**Funding acquisition:** Hongbo Chen.

**Software:** Yuancheng Gao and Yue Ji.

**Supervision:** Cheng Chen and Rui Gong.

**Writing—original draft:** Yulin Song and Rui Gong.

**Writing—review and editing:** Cheng Chen and Hongbo Chen.
